# Influence of bone morphogenetic protein (BMP) signaling and masticatory load on morphological alterations of the mouse mandible during postnatal development

**DOI:** 10.1016/j.archoralbio.2024.106096

**Published:** 2024-09-24

**Authors:** Amber Uptegrove, Coral Chen, Madison Sahagun-Bisson, Anshul K. Kulkarni, Ke’ale W. Louie, Hiroki Ueharu, Yuji Mishina, Maiko Omi-Sugihara

**Affiliations:** a Department of Biologic and Materials Sciences & Prosthodontics, University of Michigan School of Dentistry, Ann Arbor, USA; b Department of Orthodontics and Dentofacial Orthopedics, Graduate School of Dentistry, Osaka University, Osaka, Japan

**Keywords:** BMP, Mandible, Condyle, Mechanical loading, Soft diet, Mastication

## Abstract

**Objective:**

Bone homeostasis relies on several contributing factors, encompassing growth factors and mechanical stimuli. While bone morphogenetic protein (BMP) signaling is acknowledged for its essential role in skeletal development, its specific impact on mandibular morphogenesis remains unexplored. Here, we investigated the involvement of BMP signaling and mechanical loading through mastication in postnatal mandibular morphogenesis.

**Design:**

We employed conditional deletion of *Bmpr1a* in osteoblasts and chondrocytes via *Osterix*-Cre. Cre activity was induced at birth for the 3-week group and at three weeks for the 9-week and 12-week groups, respectively. The conditional knockout (cKO) and control mice were given either a regular diet (hard diet, HD) or a powdered diet (soft diet, SD) from 3 weeks until sample collection, followed by micro-CT and histological analysis.

**Results:**

The cKO mice exhibited shorter anterior lengths and a posteriorly inclined ramus across all age groups compared to the control mice. The cKO mice displayed an enlarged hypertrophic cartilage area along with fewer osteoclast numbers in the subchondral bone of the condyle compared to the control group at three weeks, followed by a reduction in the cartilage area in the posterior region at twelve weeks. Superimposed imaging and histomorphometrical analysis of the condyle revealed that BMP signaling primarily affects the posterior part of the condyle, while mastication affects the anterior part.

**Conclusions:**

Using 3D landmark-based geometric morphometrics and histological assessments of the mandible, we demonstrated that BMP signaling and mechanical loading reciprocally contribute to the morphological alterations of the mandible and condyle during postnatal development.

## 1. Introduction

Bones are constantly in a state of remodeling: old bone is removed and replaced with new bone. Intricate communication among diverse bone cell types orchestrates this equilibrium between bone formation and resorption. Evidence suggests that bone cells at distinct skeletal sites exhibit specialized functions crucial for maintaining skeletal homeostasis (Aghaloo et al., 2010; Akintoye et al., 2006; Chaichanasakul et al., 2014). Notably, the mandible differs from other skeletal bones owing to its origin from migrating cranial neural crest cells and its predominantly intramembranous ossification process, setting it apart from the axial and appendicular skeletons (Chai et al., 2000; Kronenberg, 2003; Omi & Mishina, 2020a). Anatomically, the mandible is connected to the temporal bone through the temporomandibular joint (TMJ) and plays pivotal roles in mastication, swallowing, and speech. During embryonic development, cranial neural crest cells migrate to the first branchial arch and condense to form Meckel’s cartilage, a transient structure supporting early mandibular growth before disappearing in later stages of development. The mandible’s body, formed lateral to Meckel’s cartilage, undergoes intramembranous ossification. In contrast, the mandibular condylar cartilage, a secondary cartilage, is formed after the primary cartilage forms and undergoes endochondral ossification. Notably, the condyle functions as a principal growth center for the mandible, significantly influencing postnatal mandibular growth (Mizoguchi et al., 2013).

One of the essential features influencing morphogenesis and turnover of the mandible is the mechanical stimulus incurred during mastication (Bresin, 2001; Omi & Mishina, 2020a). It has been reported that mandibular loading is associated with mandibular shape variations in human subjects (Sella-Tunis et al., 2018; Weijs, 1989; Kiliaridis et al., 1989). Similarly, studies on mice subjected to reduced masticatory load have shown consequential alterations in mandibular shape (Hassan et al., 2020; Hichijo et al., 2015). Moreover, optimal mechanical loading plays a role in promoting the proliferation and maturation of chondrocytes within the mouse condylar cartilage (Sobue et al., 2011). These studies suggest the important role of mechanical stimulation in facilitating the normal growth and turnover processes within the mandible and its condylar cartilage.

Bone morphogenetic protein (BMP) signaling plays an essential role in skeletal development and the maintenance of bone homeostasis (Kamiya & Mishina, 2011; Lin et al., 2016; Mishina et al., 2004). BMPs belong to the transforming growth factor-beta (TGF-beta) superfamily and were identified by their remarkable capacity for ectopic bone formation within soft tissues (Lowery & Rosen, 2018; Urist, 1965). These BMP ligands form either homodimers or heterodimers, engaging heterotetrameric receptors comprised of two type 1 receptors and two type 2 receptors (Wrana et al., 1994). The type 1 receptors undergo phosphorylation in their C-terminal regions upon ligand binding, specifically activating Smad1, Smad5, and/or Smad9. Subsequently, these activated Smads form complexes with Smad4, resulting in nuclear translocation and consequent modulation of gene expression (Heldin & Moustakas, 2012; Miyazawa & Miyazono, 2017). Beyond this canonical BMP-Smad signaling pathway, the ligand-receptor complexes elicit diverse non-Smad pathways, including the MAPK pathways (Zhang, 2017).

Our previous studies have demonstrated that conditional deletion of *Bmpr1a* in osteoblasts results in higher trabecular bone mass in various skeletal regions, including the skull, long bones, lumbar vertebrae, and ribs. This pronounced effect arises from an imbalance in bone remodeling, specifically diminished bone resorption (Kamiya et al., 2008a, 2008b, 2010; Zhang et al., 2016, 2020; Shi et al., 2018). Moreover, mechanical loading induced through exercise regimens synergistically augments trabecular bone volume and improves the mechanical properties of the cKO long bones (Iura et al., 2015). This finding suggests a synergistic relationship between mechanical loading and the suppression of BMP signaling, culminating in higher bone mass within long bones. However, in contrast to the discerned mechanisms in long bones, the precise cellular and molecular mechanisms underlying the impact of BMP signaling on mandibular morphogenesis, particularly its interaction with mechanical stress, remain unknown. Herein, we investigated the roles of BMP signaling in conjunction with the mechanical load by mastication in the morphological alterations of the mandible and condyle during postnatal development.

## 2. Material & methods

### 2.1. Animals

A total sample of 101 male mice were used in the study. To generate *Bmpr1a* cKO mice, we bred mice heterozygous for *Bmpr1a* carrying the Tet-off *Osterix*-*C*re (*Bmpr1a*^+/−^*;Osx-Cre*) (Mishina et al., 1995; Rodda & McMahon, 2006) with mice homozygous for the conditional allele of *Bmpr1a* (*Bmpr1a*^*fx/fx*^*;R26R/R26R*) (Mishina et al., 2002; Soriano, 1999). Mice genotyped *Bmpr1a*^*fx/*+^;*Osx-Cre;R26R*/+ and *Bmpr1a*^*fx/−*^;*Osx-Cre; R26R*/+ were designated as control and *Bmpr1a* cKO, respectively. To monitor the activity of Cre recombinase, *Osx-Cre* mice were crossed with *R26mTmG* mice (Muzumdar et al., 2007). Mice were kept in a mixed background of 129S6 and C57BL6/J. All animal experiments in this study were approved by the Institutional Animal Care and Use Committee (IACUC) at the University of Michigan (protocol #PRO00009613 and PRO00011263) and were conducted in accordance with ARRIVE guidelines.

### 2.2. Experimental groups

The sample size was calculated using G*Power3.1 with a desired power of 0.9 and a desired significance level of 0.05 based on the previous studies (Enomoto et al., 2010; Shi et al.,2018; Omi et al., 2020b). The control and cKO mice (n = 27/genotype) were randomly divided into three groups: 3-week (n = 7/genotype), 9-week (n = 10/genotype) and 12-week (n = 10/genotype) groups. For the 3-week group, Cre activity was suppressed until the day of birth by administering a diet containing 625 mg/kg doxycycline (Envigo, USA) to deliver a daily dose of 2–3 mg/mouse of doxycycline to pregnant mothers. For the 9-week group and 12-week group, Cre activity was suppressed until postnatal day 21. In both groups, Cre activity was induced by transitioning mice to a regular rodent diet either after delivery or after weaning. To understand the influence of mastication, the control and cKO mice (n = 22/genotype) at 3-week-old were provided either a solid (hard diet: HD) or powdered (soft diet: SD) diet until the collection of samples at 9-week (n = 5/group) and 12-week (n = 6/group). The powered diet was used without mixing with water. The mTmG mice were used to monitor the activity of Cre recombinase (n = 3). The body weight was measured at the end of the experiment.

### 2.3. Micro-computed tomography (micro-CT) and Image analysis

The hemi-mandibles (n = 7/group for 3-week, n = 10/group for 9-week and 12-week groups, and n = 5/group for 9-week HD/SD groups) were placed in a 19 mm diameter specimen holder and scanned over the entire length of the mandible using a micro-CT system (μCT100 Scanco Medical, Bassersdorf, Switzerland). Scan settings were voxel size 12 μm, 70 kVp, 114 μA, 0.5 mm AL filter, and integration time 500 ms. The 3D surface models of mandibles were generated based on the micro-CT data using ITK-SNAP (http://www.itksnap.org). For linear and angular analysis, landmarks were placed on the individual bone surfaces by using “modules” developed in 3D Slicer (http://www.slicer.org) (Fedorov et al., 2012). Previous studies defined landmarks’ positions (Khominsky et al., 2018; Klingenberg, 2009; Spassov et al., 2017) with certain modifications (Table 1). The 3D Slicer modules were used to superimpose the two condylar heads based on landmarks (CMF Registration) and to visualize shape differences using point-to-point alignment via computer (Shape Population Viewer) (Gazzani et al., 2018; Snider et al., 2021). The calibration process for landmark location was performed by two investigators using randomly selected images. After satisfactory calibration sessions, each image was analyzed by the trained investigator. For the subarticular bone analysis, a 75 pixel (0.9 mm) diameter circle was centered in the middle of the condyle beneath the articular surface. The region of interest (ROI) was defined as the middle of the condyle based on the first and last slice where the condyle appeared on the scan; once the middle was determined the cylinder contour was extended to 5 slices on either side of the center, for a cylinder with the dimensions of 0.9 mm D × 0.12 mm H. Bone volume (BV/TV), density (BMD) and structure model index (SMI) were calculated using a fixed global threshold of 30 % (300 on a grayscale of 0–1000).

### 2.4. Histology and histomorphometry

The hemi-mandibles (n = 7/group for 3-week and n = 6/group for 12-week HD/SD groups) were fixed in 4 % paraformaldehyde, decalcified using 10 % EDTA, and subsequently embedded in paraffin. After the whole condyle had been series sectioned (7 μm thickness) along the anterior-posterior (A-P) axis, three sections that corresponded to the central mediolateral area of the condyle were stained according to standard protocols for hematoxylin and eosin (H&E), safranin O, toluidine blue, and tartrate-resistant acid phosphatase (TRAP) stains. Histomorphometric analysis of the condylar cartilage was conducted using ImageJ/Fiji. The mandibular condylar cartilage was divided into fibrous and proliferating, prehypertrophic, and hypertrophic cell layers based on cell shape, size, and staining intensity (Liu et al., 2022). The heights of each layer were measured at three locations: posterior, central, and anterior points, and plotted as ratios. The central point of the condyle was defined as the point of tangency of a line parallel to the line between the most anterior and posterior points of the condylar head. Heights of each layer were measured along a line perpendicular to the tangent. The anterior and posterior points were determined as follows: First, the midpoints between the most anterior side and the central point were determined along the surface of the condylar head and the boundary between cartilage and bone. Second, a line was drawn between these midpoints, and the heights of each layer were measured. Similar steps were taken to determine the location of the posterior points. Details are provided in Fig. 4B. Three sections from each animal (n = 7/group for 3-week and n = 6/group for 12-week HD/SD groups) were measured to calculate averages. The areas of hypertrophic cartilage area and heights of each layer were measured by one investigator. For the mTmG mice (n = 3), condylar heads were embedded in OCT, and a series of 10 μm sagittal sections were prepared and mounted with ProLong Gold anti-fade reagent, including with 4′,6-diamidino-2-phenylindole (DAPI) (Invitrogen).

### 2.5. Statistical analysis

Data were analyzed with GraphPad Prism 9.0 software (GraphPad Software, Boston, Massachusetts, USA). Statistical differences between the two groups were analyzed using Mann-Whitney test, and those among four groups were analyzed using a Kruskal-Wallis test followed by a Dunn’s multiple comparison post-hoc test. The significance level was obtained for all tests when p-value is < 0.05. The data is presented in a Box-and-Whisker plot format. The upper and lower boundaries of the box represent the 75th and 25th percentiles, respectively. The line inside the box depicts the 50th percentile (median), while the whiskers extend to show the range.

## 3. Results

### 3.1. Osx-Cre marks osteoblasts, chondrocytes, and progenitor cells within the condyle

**Fig. 1.**
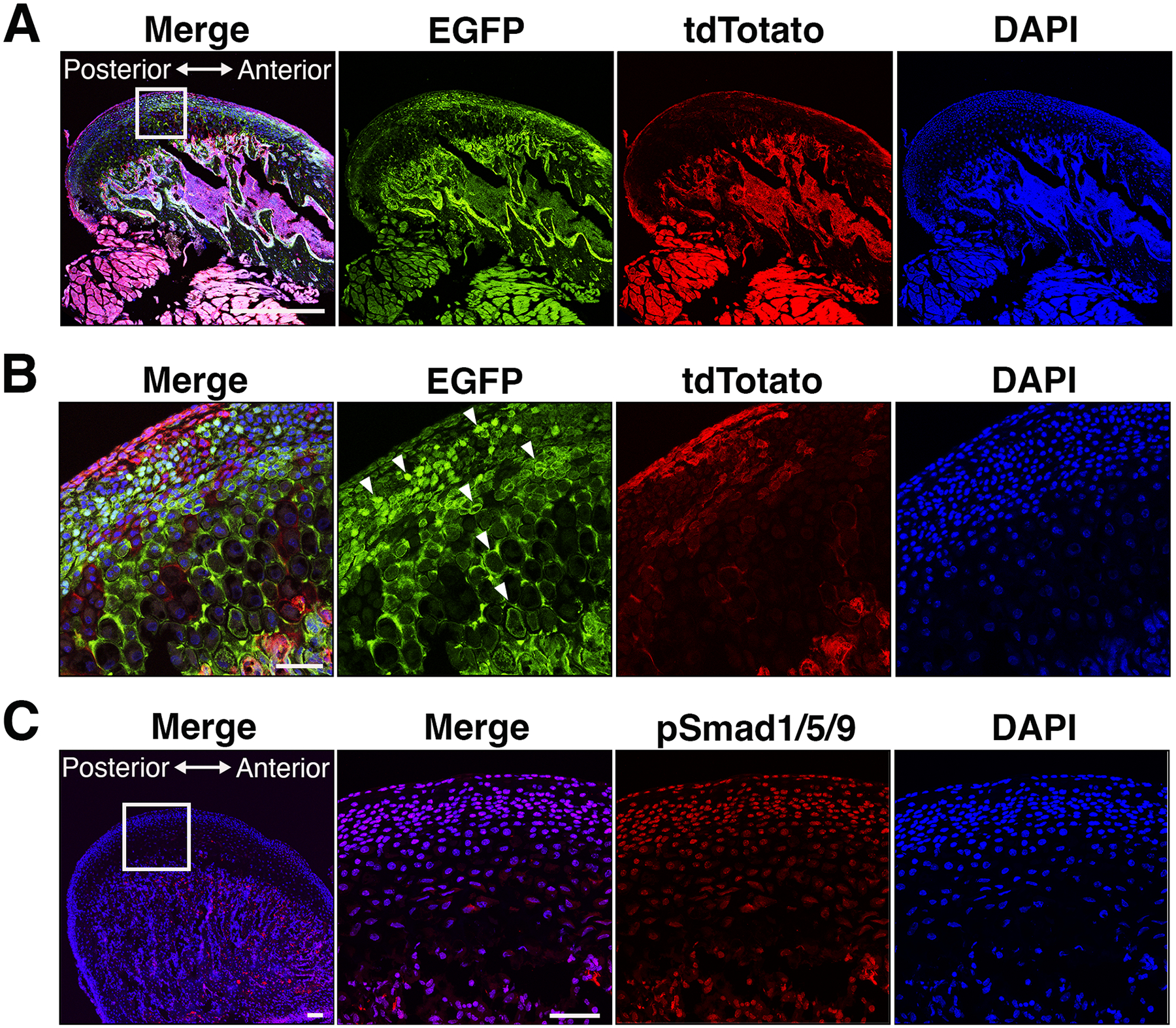
*Osterix-Cre* marks chondrocytes and progenitor cells within the mandibular condyle. Lower (**A**) and higher (**B**) magnification images of the mandibular condylar head of *Osterix-Cre*;mTmG mice at 3 weeks of age. The transmembrane form of tdTotato expression shifts to the transmembrane form of GFP expression after recombination. Cre activity was activated at birth by switching from doxycycline chow to the regular rodent diet of nursing mothers. Arrows indicate *osterix*-expressing chondrocytes and progenitor cells. (**C**) Lower and higher magnification images of the mandibular condylar head stained with p-Smad1/5/9 at 3 weeks of age. Scale = 500 μm (A), 50 μm (B), 100 μm (C).

We observed the embryonic lethality resulting from the conditional disruption of *Bmpr1a* using constitutively active *Osx-Cre* (data not shown). Thus, we implemented an inducible Tet-off system to activate Cre activity during postnatal development. In a previous report, we demonstrated that the removal of doxycycline chow at birth effectively induces Cre recombinase activity in odontoblasts of the teeth and osteoblasts within the mandibular alveolar bone at 3 weeks (Omi et al., 2020b). Given that *osterix* expresses in chondrocytes during endochondral ossification (Fedorov et al., 2012; Jing et al., 2014a, 2014b), we investigated whether Cre recombination occurred efficiently in the chondrocytes within the mandibular condylar cartilage using mTmG Cre reporter mice. Our analysis confirmed the presence of GFP-positive cells within the proliferative, prehypertrophic and hypertrophic layers of the condylar cartilage as well as the subchondral bones at 3 weeks of age when Cre activity was induced at birth (Fig. 1A, B). These findings suggest that *Osx-Cre* is expressed in both osteoblasts and chondrocytes within the mandibular condyle. To understand BMP signaling activity in condyle, the levels of phosphorylated forms of Smad1/5/9 (p-Smad1/5/9) were assessed by immunohistochemistry (Fig. 1C). The p-Smad1/5/9 was observed in the nuclei of proliferative, prehypertrophic and hypertrophic chondrocytes within the condyle at 3 weeks of mice.

### 3.2. Disruption of BMP signaling in osteoblasts and chondrocytes results in alterations in mandibular morphology

**Fig. 2.**
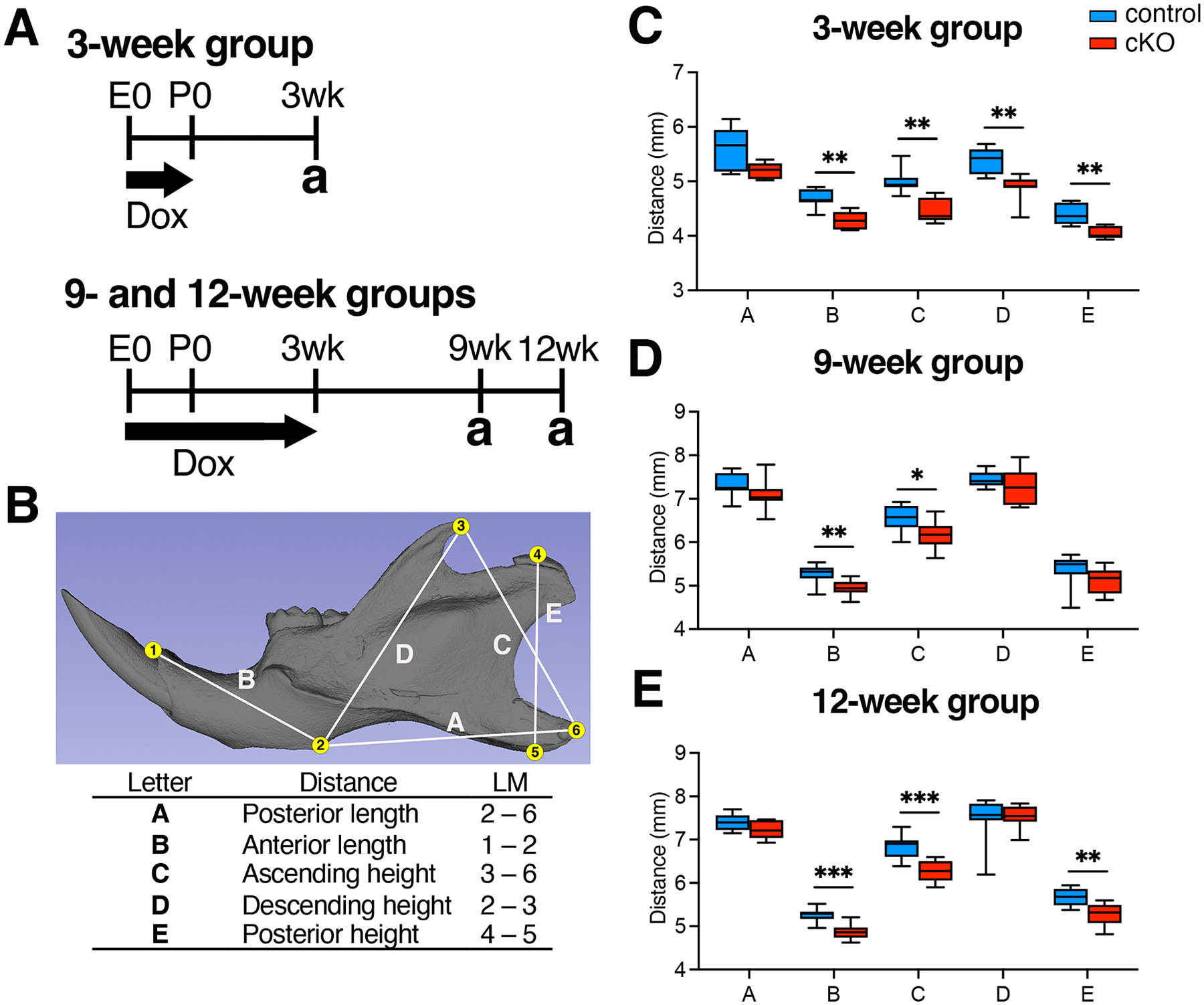
The loss of BMP signaling leads to significant alterations in the size and shape of the mandible. (**A**) The schematic illustrates the experimental design. Recombination was prevented through the administration of doxycycline (Dox) administration starting from embryonic day 0 (E0). For the 3-week group, Cre activity was activated at postnatal day 0 (P0) by transitioning from doxycycline chow to the regular rodent diet provided to nursing mothers. Analysis (a) was conducted at 3 weeks of age. In the 9- and 12-week groups, Cre activity was activated at 3 weeks by transitioning from doxycycline chow to the regular rodent diet. Analysis was conducted at 9 and 12 weeks of age. (**B**) An isosurface representation of a hemi-mandible displaying six landmarks (yellow dots) and five linear measurements (white lines) used in the study: posterior length (2—6), anterior length (1—2), ascending height (3—6), descending height (2—3), and posterior height (4—5). Linear measurements of the mandible in control and *Bmpr1a* cKO mice were taken at 3 weeks (**C**), 9 weeks (**D**), and 12 weeks (**E**). LM, landmark, n = 7 each (3-week), 10 each (9-week), 10 each (12-week). *p<0.05, **p<0.01, ***p<0.001.

To investigate the impact of BMP signaling on postnatal mandibular growth and morphogenesis, we conducted a three-dimensional (3D) morphological analysis of mouse mandibles using micro-CT imaging. Research has demonstrated a significant decline in growth velocity in various cranial and facial bones, including the mandible of C57BL/6 J mice, notably by postnatal day 21. This decline continues, reaching values near zero by 12 weeks (Vora et al., 2015). Consequently, our study aimed to explore the impact of BMP signaling on mandibular morphogenesis during both early and late postnatal mandibular development. In detail, Cre activity was induced at birth for the 3-week group to evaluate early postnatal mandibular growth. For the 9- and 12-week groups, Cre activity was induced at 3 weeks to assess late postnatal mandibular growth (Fig. 2A). The body weights of each group at 3, 9 and 12-weeks showed no change at the end of the experiment (Supplementary Figure 1A–C). Three-dimensional coordinate locations of six mandibular landmarks were used for a linear analysis of the mandible (Fig. 2B). Five linear measurements were subsequently evaluated to assess mandibular alterations: posterior length (2—6), anterior length (1—2), ascending height (3—6), descending height (2—3), and posterior height (4—5). Compared with the control mandibles, cKO mandibles in the 3-week group exhibited the following trends: (i) a significantly shorter anterior length (ii) significantly lower descending, ascending, and posterior heights (Fig. 2C). There were no significant differences in the ratio of each measurement (Supplementary Figure 2A). This suggests that all absolute lengths were proportionally smaller, indicating a reduced size of the mandible in the cKO mice. In the 9-week group, cKO mandibles exhibited (i) a significantly shorter anterior length and (ii) a significantly lower ascending height but unaltered descending and posterior heights (Fig. 2D). The ratio of ascending height to posterior length is significantly smaller in the cKO mice, whereas the ratio of descending height to ascending height is notably higher in the cKO mice (Supplementary Figure 2B**, measurements C/A & D/C**). This implies a notable reduction in ascending height among the cKO mice. In the 12-week group, cKO mandibles exhibited (i) a significantly shorter anterior length and (ii) significantly lower ascending and posterior heights with an unaltered descending height (Fig. 2E). The ratios of anterior length, ascending height, descending height and posterior height to posterior length were smaller in the cKO mice (Supplementary Figure 2C**, measurements B/A, C/A, D/A & E/A**). Additionally, the ratio of posterior height to descending height was smaller in the cKO mice (Supplementary Figure 2C**, measurements E/D**). Conversely, the ratios of descending height to anterior length and descending height to ascending height were larger in the cKO mice (Supplementary Figure 2C**, measurements D/B & D/C**). These data demonstrate a notable proportional difference in the shape of the mandible between the cKO mice and the control group as they age.

**Fig. 3.**
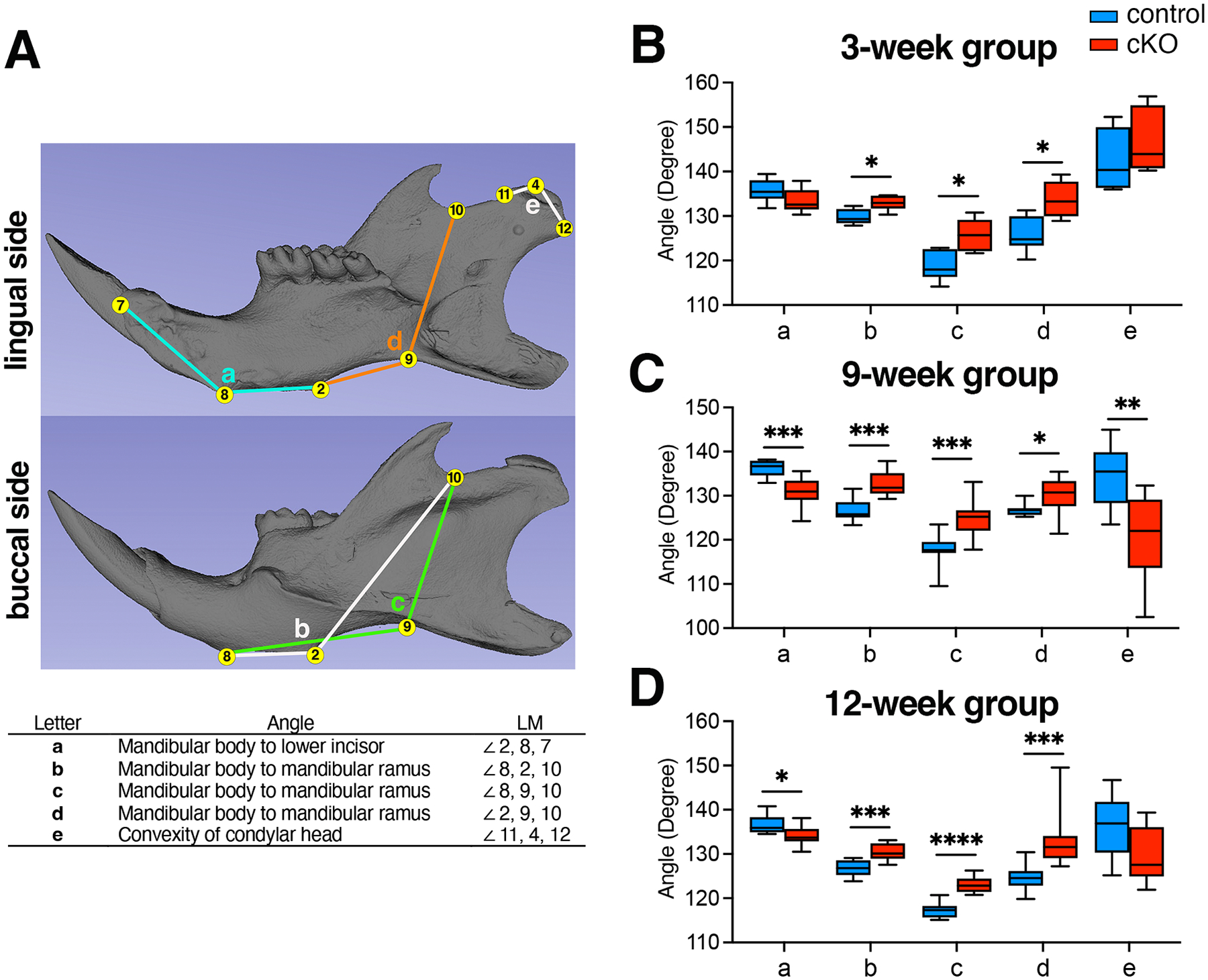
The loss of BMP signaling results in significant alterations in the inclination of the ramus and the overall shape of the mandibular condyle. (**A**) The isosurface representation of a hemi-mandible displaying eight landmarks (yellow dots) and five angular measurements (color lines) used in the study: mandibular body to lower incisor (2—8—7), mandibular body to ramus (8—2—6, 8—9—10, 2—9—10), and convexity of the condylar head (11—4—12). Angular measurements of the mandible in control and *Bmpr1a* cKO mice were taken at 3 weeks (**B**), 9 weeks (**C**), and 12 weeks (**D**). LM, landmark, n = 7 each (3-week), 10 each (9-week), 10 each (12-week). *p<0.05, **p<0.01, ***p<0.001, ****p<0.0001.

For angular analysis, we utilized 3D coordinate locations of eight landmarks (Fig. 3A). Five angular measurements were subsequently evaluated to assess the growth direction of the incisor and ramus, as well as the convexity of the condyle: mandibular body to lower incisor (2—8—7), mandibular body to ramus (8—2—6, 8—9—10, 2—9—10), and convexity of the condylar head (11—4—12). Compared with control mandibles, cKO mandibles in the 3-week group exhibited: (i) an unaltered angle of the mandibular body to the lower incisor, (ii) a significantly greater angle of the mandibular body to the mandibular ramus, (iii) an unaltered convexity of the condylar head (Fig. 3B). In the 9-week group, cKO mandibles exhibited (i) a significantly smaller angle of the mandibular body to the lower incisor, (ii) a significantly greater angle of the mandibular body to the mandibular ramus, (iii) higher convexity of the condylar head (Fig. 3C). In the 12-week group, cKO mandibles exhibited (i) a significantly smaller angle of the mandibular body to the lower incisor, (ii) a significantly greater angle of the mandibular body to the mandibular ramus, (iii) an unaltered convexity of the condylar head (Fig. 3D). These findings underscore the crucial role of BMP signaling in mandibular and condylar head morphogenesis.

### 3.3. Disruption of BMP signaling in osteoblasts and chondrocytes results in thicker hypertrophic layers at 3 weeks

**Fig. 4.**
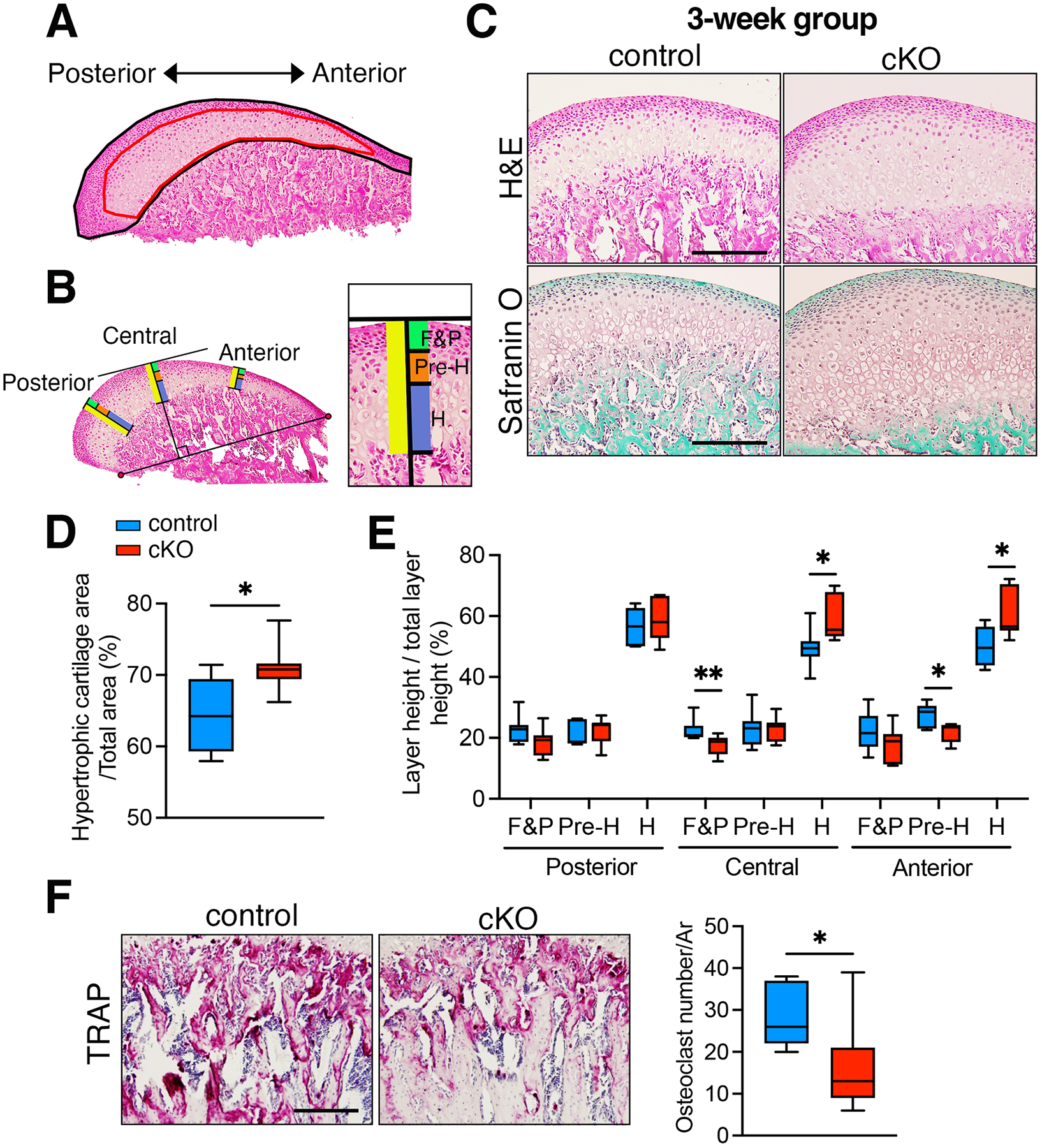
The loss of BMP signaling leads to a larger hypertrophic cartilage area at 3 weeks. (**A**) Diagram illustrates the measurements of hypertrophic cartilage area (red) against the total area (black) within the mandibular condyle. (**B**) The mandibular condylar cartilage was divided into fibrous and proliferating (green), prehypertrophic (orange), and hypertrophic (blue), and total (yellow) cell layers based on cell shape, size, and staining intensity. The heights of each layer were measured at three locations: posterior, central, and anterior points, and plotted as ratios. The central point of the condyle was defined as the point of tangency of a line parallel to the line between the most anterior and posterior points of the condylar head. Heights of each layer were measured along a line perpendicular to the tangent. The anterior and posterior points were determined as follows: First, the midpoints between the most anterior side and the central point were determined along the surface of the condylar head and the boundary between cartilage and bone. Second, a line was drawn between these midpoints, and the heights of each layer were measured. Similar steps were taken to determine the location of the posterior points. (**C**) Representative sections of the central mediolateral area of the condyle, stained with H&E and Safranin O staining, from both control and *Bmpr1a* cKO mice at 3 weeks are presented. Scale = 200 μm. (**D**) The hypertrophic cartilage area relative to the total area was compared between control and *Bmpr1a* cKO mice. (**E**) The percentage of fibrous and proliferative (F&P), pre-hypertrophic (Pre-H) and hypertrophic (H) chondrocyte layer relative to the total layer in the anterior, central and posterior regions of the condyle was compared between control and *Bmpr1a* cKO mice. (**F**) The number of osteoclasts in the subchondral bone of the condyle was counted using TRAP staining in both control and *Bmpr1a* cKO mice. n = 7, *p<0.05, **p<0.01.

Following our observations of reduced BMP signaling leading to alterations in the shape of the condylar head through micro-CT investigation, we extended our analysis to explore its impact on condylar cartilage formation via histological examination. At 3 weeks of age, we measured the hypertrophic cartilage area/total area and heights of distinct cartilage layers of the anterior, central, and posterior parts/total heights of the condylar cartilage (Fig. 4A, B). Employing H&E and safranin O stains, our analysis revealed a significantly larger hypertrophic cartilage area in the cKO condyle compared to the controls at 3 weeks of age (Fig. 4C, D). Notably, the hypertrophic chondrocyte layer of the anterior and central condylar parts of the cKO mice exhibited a significant thickening, resulting in a larger cartilage area within the mandibular condyle (Fig. 4E). Osteoclast activity of the subchondral bone of the condyle was assessed by TRAP staining. The condylar subchondral bone of the cKO mice exhibited lower osteoclast numbers compared to control (Fig. 4F). These findings suggest that reduced BMP signaling in osteoblasts and chondrocytes contributes to condylar cartilage formation and configuration, and subchondral bone remodeling during early postnatal development.

### 3.4. Reduced masticatory load leads to morphological alterations in the mandibular condyle

**Fig. 5.**
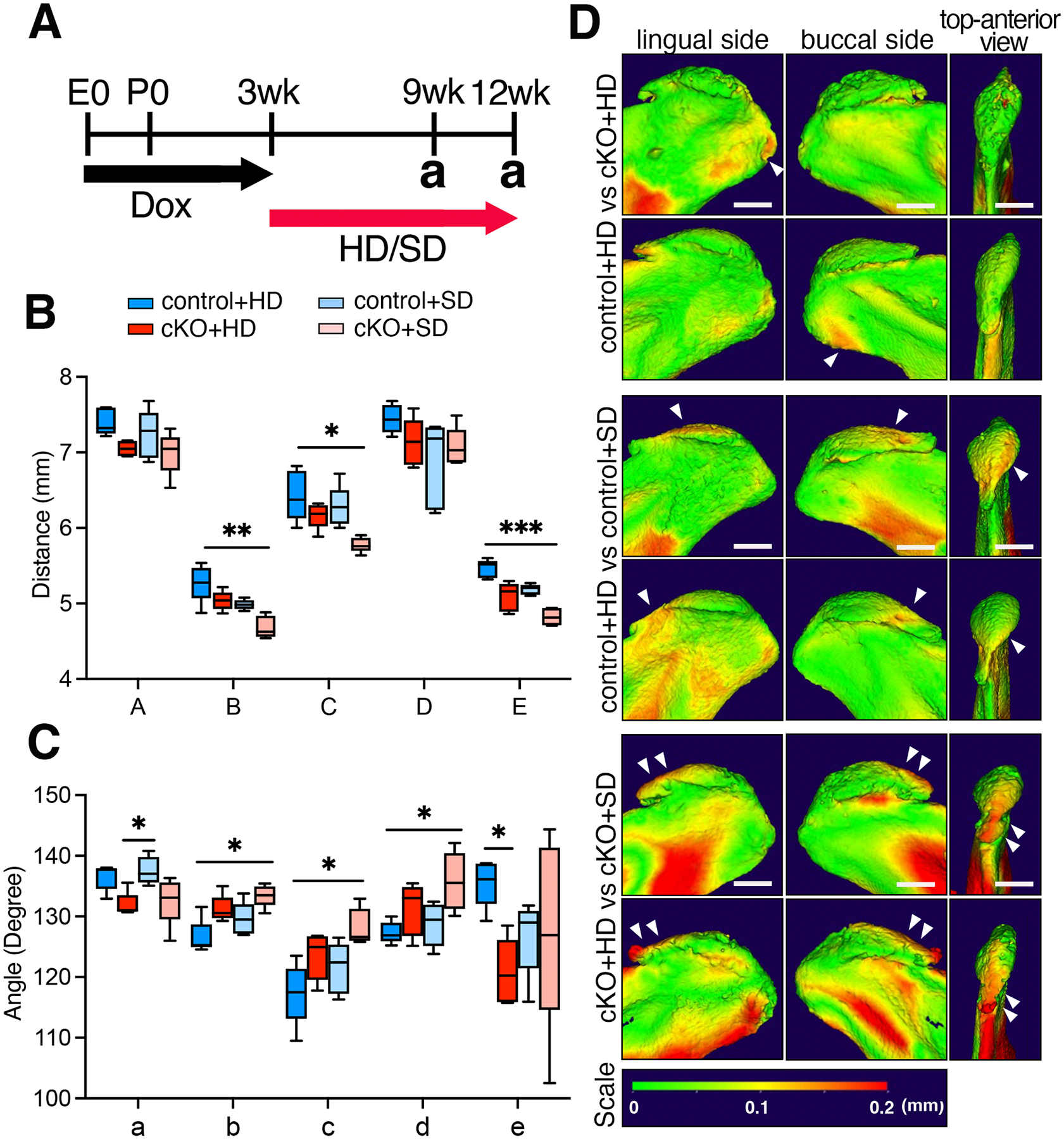
The loss of BMP signaling and a soft diet regimen leads to significant alterations in the size and shape of the mandible and condyle at 9 weeks. (**A**) Schematic illustrates the experimental design. Recombination was prevented through the administration of doxycycline (Dox) administration starting from embryonic day 0 (E0). Cre activity was activated at 3 weeks by transitioning from doxycycline chow to either the regular rodent diet (hard diet: HD) or the powdered diet (soft diet: SD). Analysis (a) was conducted at 9 and 12 weeks of age. Mandibular measurements of distances (**B**) and angles (**C**) were taken in both the control and *Bmpr1a* cKO mice at 9 weeks. A-E in panel B, length measurements described in Fig. 2B, a-e in panel C, angle measurements described in Fig. 3A. (**D**) Representative superimposing images of the mandibular condylar head from the control and cKO mice at 9 weeks. Two typical images from each group are shown. The color gradient indicates the differences in bone surface distances between the two groups. Arrows indicate a large difference. Scale = 500 μm. n = 5, *p<0.05,**p<0.01, ***p<0.001.

To investigate the effect of mechanical load induced by mastication on mandibular and condylar cartilage formation, mice were subjected to either a regular diet (hard diet, HD) or a powdered diet (soft diet, SD) for 6 weeks (9-week group) or 9 weeks (12-week group) after the removal of doxycycline chow at 3 weeks of age (Fig. 5A). The body weights of each group fed either HD or SD for 6 weeks showed no change at the end of the experiment (Supplementary Figure 1D). The cKO+SD group exhibited a shorter anterior length, and lower ascending and posterior heights compared to the control+HD group (Fig. 5B, **measurements B, C, E and** Supplementary Figure 2D**, measurement B/A, C/A, D/B, D/C**). Regarding angular analysis, the cKO+SD group exhibited a greater angle of the mandibular body to the mandibular ramus compared to the control+HD group (Fig. 5C, **measurement b, c, d**). To visualize regional alterations in shape and scaling of the mandibular condyle, we digitally superimposed control surface models with corresponding cKO or HD/SD samples (Fig. 5D). The superimposing control groups exhibited no overt changes (Supplementary Figure 3). Significant differences in the posterior region of the condylar head were detected between the control+HD and cKO+HD groups. On the other hand, significant differences in the anterior region of the condylar head were observed between the control+HD and control+SD groups. Similar deviations in the anterior region of the condylar head were also noted in the comparison between cKO+HD and cKO+SD groups, with the deviations being more pronounced than those between control+HD and control+SD groups. These findings suggest that BMP signaling and mechanical loading affect distinct condyle regions differently. Moreover, these factors likely collaborate in regulating the anterior morphogenesis of the mandibular condyle.

### 3.5. Soft diet feeding leads to a larger condylar cartilage area in both control and Bmpr1a cKO mice at 12 weeks

**Fig. 6.**
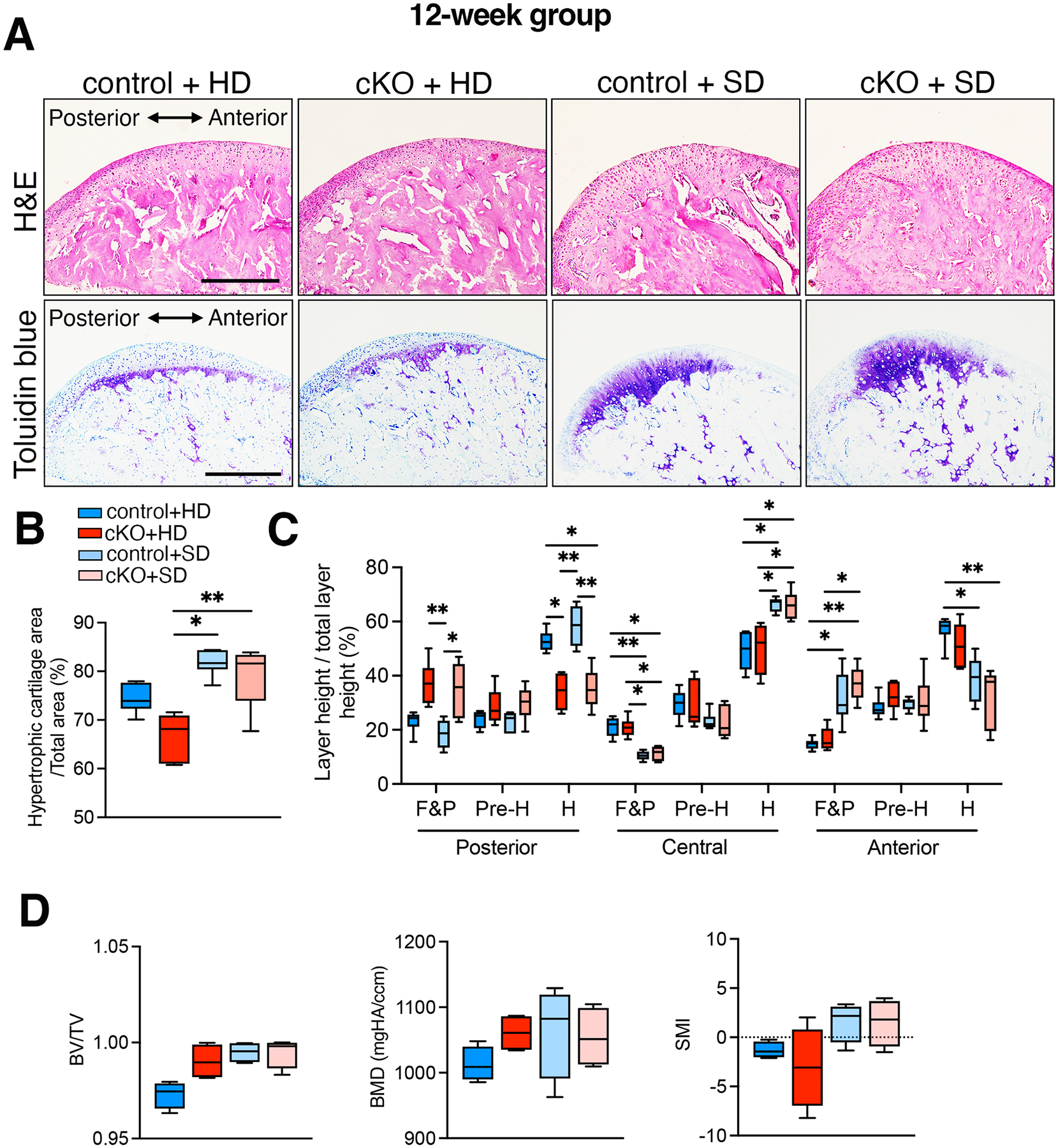
Soft diet feeding results in a grater hypertrophic cartilage area in both control and *Bmpr1a* cKO mice at 12 weeks. Representative central mediolateral sections displaying H&E and toluidine blue staining in control and *Bmpr1a* cKO mice at 12 weeks. Scale = 200 μm. (**B**) The hypertrophic cartilage area against the total area, as shown in Fig. 4A, was compared between the control and *Bmpr1a* cKO mice fed with the regular rodent diet (hard diet: HD) or the powdered diet (soft diet: SD). (**C**) The heights of the fibrous and proliferative layer (F&P), prehypertrophic layer (Pre-H), and hypertrophic layer (H) against the total layer, as shown in Fig. 4B, were measured. (**D**) Bone volume (BV/TV), density (BMD) and structure model index (SMI) in the subchondral bone of the condyle were measured. n = 6, *p<0.05, **p<0.01.

To investigate the impact of mechanical load on mandibular condylar cartilage formation, we analyzed central mediolateral sections stained with H&E and toluidine blue of both *Bmpr1a* cKO and control mice at 12 weeks (Fig. 6A). These mice were subjected to either a hard diet or a soft diet for 9 weeks after the removal of doxycycline chow at 3 weeks of age. In contrast to the 3-week group, the 12-week cKO mice exhibited a tendency for a smaller hypertrophic cartilage area compared to the age-matched control mice (Fig. 6B). In control mice, the hypertrophic cartilage area extended uniformly from the anterior to the posterior of the condyle. In contrast, in cKO mice, the hypertrophic cartilage area was expanded in the central region of the condylar head, accompanied by a substantial reduction of hypertrophic chondrocyte layer in the posterior region (Fig. 6C). Notably, a reduction in hypertrophic chondrocytes accompanied by more significant cell numbers was evident in the posterior region of the cKO condyle. These findings suggest that BMP signaling plays a role in regulating cartilage formation and its distribution along the anterior-posterior axis in the condyle head. Interestingly, both control and cKO mice on a soft diet for 9 weeks displayed a larger hypertrophic cartilage area and a thickened hypertrophic chondrocyte layer in the central part of condyle compared to their corresponding mice on a hard diet. While proportion of each cartilage layer was not affected by a soft diet in the posterior region of condyle, soft diet feeding significantly reduced the anterior region of hypertrophic chondrocyte layer both in control and cKO mice. These findings demonstrate that both BMP signaling and mechanical loading by mastication influence condylar cartilage formation. Analyses of the cartilage distribution pattern suggest that BMP signaling primarily affects the posterior part of the condyle, while mastication affects the anterior part. For the subchondral bone of condyle, there was no statistically significant difference in BV/TV, BMD or SMI, both control and cKO mice on a soft diet displayed a tendency for higher BV/TV, BMD and SMI compared to their corresponding mice on a hard diet (Fig. 6D).

## 4. Discussion

In this study, we utilized *Osx-Cre* to delete *Bmpr1a* in osteoblasts and chondrocytes within the mandible, aiming to understand the role of BMP signaling and mechanical load induced by mastication during postnatal mandibular development. Our analysis of micro-CT images and histological sections allowed us to discern alterations in the size and shape of the mandible resulting from reduced BMP signaling and changes in mechanical load through mastication. Linear analysis revealed that cKO mice exhibited a shorter anterior length and a lower posterior height compared to control mice. These findings underscore the important role of BMP signaling in both anterior and posterior mandibular growth. Angular analysis revealed that cKO mice exhibited a posterior inclination of the ramus and a lingual inclination of the lower incisor compared to control mice, indicating the involvement of BMP signaling in determining the growth direction of the mandibular ramus and the incisor. Analysis of the condylar cartilage indicated that cKO mice displayed a larger hypertrophic cartilage area at 3 weeks. However, a reduced area was observed in the posterior region of the condyle at 9 weeks, suggesting that BMP signaling exerts distinct influences on the formation of condylar cartilage across various developmental stages. Moreover, mechanical loading induced by mastication resulted in shape alterations of the anterior condyle, attributed to changes in cartilage area and configuration. This finding underscores the cooperative regulation of morphogenesis in the mandibular condyle by BMP signaling and mechanical loading. Overall, our findings highlight the essential role of BMP signaling in both mandibular and condylar cartilage morphogenesis during postnatal development.

An important aspect to consider when analyzing postnatal morphological alterations in the mandible is the coordinated growth of the maxilla and mandible (Vora et al., 2015). We reported that the constitutive activation of *Bmpr1a* in neural crest cells results in the deformation of the cranial base, primarily due to the premature fusion of the intersphenoidal synchondrosis (Ueharu et al., 2023). While the effects of BMP signaling loss of function on maxilla and cranial base synchondrosis during postnatal development remain uncertain, a reduction of BMP signaling in osteoblasts and chondrocytes during postnatal growth could potentially lead to deformation of the maxilla and cranial base. This alteration may consequently influence the anterior and posterior growth, as well as the growth direction of the mandible in the cKO mice, although this cannot be conclusively ruled out. Moreover, we and others have reported the critical involvement of BMP signaling in tooth formation (Li et al., 2011; Omi et al., 2020b; Zhang et al., 2019), raising the possibility that impaired tooth formation due to loss of BMP signaling, particularly in the mouse incisor, might have a secondary impact on the overall shape of the mandible. In particular, a smaller angle between the mandibular body and the lower incisor could be attributed to malformation of the incisor resulting from reduced BMP signaling. Subsequent efforts will focus on elucidating alterations in other craniofacial structures resulting from the absence of BMP signaling and understanding their respective contributions to overall mandibular morphogenesis during postnatal development.

The mandibular condylar cartilage is recognized as a secondary cartilage, distinct from the primary cartilage found in the limbs and Meckel’s cartilage. Specifically, the condyle is a crucial growth center for the mandible (Mizoguchi et al., 2013). We previously reported that the postnatal deletion of *Bmpr1a* in chondrocytes using *Aggrecan-CreERT* on postnatal day 3 resulted in chondrodysplasia and reduced mandibular size by 4 weeks (Jing et al., 2014a, 2014b). This finding emphasizes the important role of BMP signaling in forming the condylar cartilage during early postnatal development. In this study, however, we found that disrupting BMP signaling in osteoblasts and chondrocytes at birth using *Osx-Cre* led to a wider hypertrophic cartilage area in the condyle compared to the control mice at 3 weeks. There are several potential reasons to account for this observation. Given that *Osx-Cre* targets both osteoblasts and chondrocytes, while *Aggrecan-Cre* primarily targets chondrocytes, the augmented cartilage area in cKO mice at 3 weeks likely stems from the impact of BMP signaling in osteoblasts. Our previous reports have indicated that the disruption of BMP signaling in osteoblasts results in impaired osteoclast formation, resulting in higher bone mass in long bones (Kamiya et al., 2008a, 2008b, 2010; Zhang et al., 2016, 2020; Shi et al., 2018). In this study, we also demonstrated that the cKO mice exhibited less osteoclast numbers in the subchondral bone of condyle at 3 weeks of age. These findings suggest that BMP signaling in osteoblasts plays a critical non-cell autonomous role in osteoclastic bone resorption. Studies have shown that the degradation of hypertrophic cartilage by osteoclast-like cells, known as chondroclasts, is essential to establish the bone trabecular network during long bone growth (Bromley & Woolley, 1984; Touaitahuata et al., 2014). Hence, the reduced BMP signaling in osteoblasts might impede cartilage resorption by osteoclasts, resulting in more hypertrophic chondrocytes within the cKO condyle during early postnatal development.

On the other hand, disrupting BMP signaling at 3 weeks reduced hypertrophic condylar cartilage area at 12 weeks in the cKO mice, suggesting that the involvement of chondroclasts in the degradation of hypertrophic cartilage may not be as significant at this later stage. Furthermore, we induced Cre activity at different time points: at birth for the 3-week group and at 3 weeks of age for the 12-week group. This differential timing likely targeted distinct cellular populations within the condylar cartilage. Finally, loading through mastication likely influences hypertrophic cartilage in the cKO mice of the 12-week group, as evidenced by the absence of a significant difference between the control+SD and cKO+SD groups. Future follow-up studies will investigate whether alterations in the condylar cartilage resulting from the loss of BMP signaling are attributed to osteoblasts, chondrocytes, or mechanical loading, aiming to understand the unique role of BMP signaling in condylar cartilage formation.

Several experimental studies have demonstrated that reducing masticatory load through soft or liquid diets leads to thinner condylar cartilage (Chen et al., 2009; Hichijo et al., 2014; Yamada & Kimmel, 1991; Yan et al., 2021; Yu et al., 2019). On the other hand, diminished masticatory function in muscular dystrophic mice results in thicker cartilage (Ghafari & Cowin, 1989). In this study, both control and cKO mice on a soft diet exhibited a larger hypertrophic cartilage area than the corresponding mice on a hard diet. Discrepancies between our findings and previous studies might stem from variations in condylar topography, animal species, age, and food consistency. Previous research has highlighted regional variations in condylar cartilage thickness. For instance, the condylar cartilage becomes thinner in the anterior part and thicker in the posterior section with a soft diet (Kiliaridis et al., 1999; Yu et al., 2019). In agreement with this finding, our study demonstrated that both the control and cKO mice on a soft diet exhibited a thinner hypertrophic chondrocyte layer in the anterior region and a thicker hypertrophic chondrocyte layer in the central part of the condyle compared to their corresponding mice on a hard diet. Interestingly, BMP signaling primarily affected the posterior region of the condylar cartilage. These findings suggest that chondrocytes in the different regions of the condyle respond differently to BMP signaling and mechanical loading during mastication. Additionally, in reported studies, hard diet groups often receive conventional hard pellet diets, while soft diet groups receive the same diet in powdered form, with or without mixing with water, altering food hardness and viscosity and influencing chewing patterns and muscular activity (van der Bilt & Abbink, 2017). Moreover, there are discrepancies in terminology and classification of the condylar cartilage layers among reported studies (Mizoguchi et al., 2013; Scheidegger et al., 2018). Thus, standardization of the method for employing different food consistencies, localization and orientation of sections used for histological evaluation, and terminology and classification in the study are valuable for understanding the impact of mechanical load on the condylar cartilage.

In addition to bone shape, both bone quantity and bone quality are important parameters in determining the properties and mechanical functions of the bone, contributing to the establishment of the treatment plan in the mandible. In the temporomandibular joint (TMJ), the cartilage mitigates stress on the condylar surface, while the subchondral bone provides structural support to the cartilage during mastication. Our study demonstrated that the cKO mice displayed a tendency for higher BV/TV in the subchondral bone of the condyle at 12 weeks, likely due to diminished bone resorption. This conclusion is supported by findings from the 3-week group, where the number of osteoclasts was significantly lower in the cKO subchondral bone. We have reported that the loss of BMP signaling in osteoblasts results in higher bone mass, partly due to diminished osteoclastic bone resorption through reduced RANKL/OPG signaling (Kamiya et al., 2008a, 2008b, 2010). While we did not assess the levels of RANKL/OPG, we anticipate observing a reduction in RANKL/OPG levels in the cKO mice. Future investigations will prioritize elucidating the molecular mechanisms to gain deeper insights into the role of BMP signaling and mechanical loading within the mandibular condyle. Surprisingly, we also observed a tendency for higher BV/TV both in the control and cKO mice on a soft diet compared to control mice on a hard diet. Generally, appropriate functional loading such as exercise improves bone quantity and quality (Duncan & Turner, 1995; Kohn et al., 2009; Iura et al., 2015). It has been reported that altered compressive loading in the subchondral bone of the condyle increases bone mass, while a reduction in loading due to a soft diet leads to decreased bone density and bone formation (Kaul et al., 2016; Bouvier & Hylander 1982; Sasaguri et al., 1998). Conversely, a study has reported an increase in osteoclasts on the chewing side and an increase in osteoblasts on the non-chewing side during long-term unilateral mastication (Liu et al., 2022). Our study demonstrated that both the control and cKO mice exhibited positive SMI values on a soft diet, while negative values were observed for both groups on a hard diet. While there is still a debate on how to corelate SMI to bone physiology, a positive SMI indicates rod-like structures (SMI > 2) in trabecular bone, and the plate-to-rod transition is an important parameter in bone loss (Salmon et al., 2015). Although we observed that the reduction of loading via a soft diet results in a trend toward higher bone quantity in the subchondral bone of the condyle, it may alter bone micro-architecture, shifting from a plate-like trabecular structure to a rod-like configuration, which is associated with osteoporosis. Needless to say, future investigations will more focus on addressing the impact of BMP signaling and mechanical loading on alterations in both the quality and quantity of bone in the mandible.

## 5. Conclusion

Using 3D landmark-based geometric morphometrics applied to the mandible and histological analysis of the condylar cartilage, noteworthy distinctions were observed among specific landmarks in the cKO and control mice. These findings signify the involvement of BMP signaling in forming both the mandible and the condylar cartilage throughout postnatal development. Furthermore, our investigation presented compelling evidence associating BMP signaling with mechanical loading induced by mastication in the formation of the condylar cartilage. Because BMP signaling and mechanical loading are associated with the onset and progression of TMJ disorders (Bechtold et al., 2016; Ikeda et al., 2014; Shirakura et al., 2017), our discoveries contribute to a deeper understanding of TMJ disorders and their potential treatment strategies.

## Supplementary Material

Supple Fig 1-3

## Figures and Tables

**Table 1:** Description of landmarks for linear and angular measurements.

Letter	Landmarks
**1**	Highest point of the alveolar ridge of the incisor
**2**	Most prominent posterio-inferior point on mental process
**3**	Most prominent point of the coronoid process
**4**	Most superior point of the condylar process
**5**	Most prominent point on inferior margin of angular process
**6**	Most prominent point on tip of angular process
**7**	Most rostral and dorsal point of the mandibular symphysis
**8**	Most prominent antero-inferior point on mental process
**9**	Deepest point on inferior border of the ramus
**10**	Lowest point of the mandibular notch
**11**	Most rostral point of the condylar process
**12**	Most caudal point of the condylar process
