# Supplementary material for: Influence of bone morphogenetic protein (BMP) signaling and masticatory load on morphological alterations of the mouse mandible during postnatal development
Source: Arch Oral Biol. Author manuscript; Available in PMC 2025 Jul 1. (PMC11609011; doi:10.1016/j.archoralbio.2024.106096)
Supplement: Supple Fig 1-3 [file NIHMS2030408-supplement-Supple_Fig_1-3.docx]

**Supplementary File**

**Influence of Bone Morphogenetic Protein (BMP) Signaling and Masticatory Load on Morphological Alterations of the Mouse Mandible During Postnatal Development.**

Running title: BMP and Mandible

Amber Uptegrove^1^, Coral Chen^1^, Madison Sahagun-Bisson^1^, Anshul K Kulkarni^1^, Ke’ale W Louie^1^, Hiroki Ueharu^1^, Yuji Mishina^1,*^, and Maiko Omi-Sugihara^1, 2,*^

1. Department of Biologic and Materials Sciences & Prosthodontics, University of Michigan School of Dentistry, Ann Arbor, USA

2. Department of Orthodontics and Dentofacial Orthopedics, Graduate School of Dentistry, Osaka University, Osaka, Japan

Corresponding authors:

Maiko Omi-Sugihara, DDS, PhD

Department of Orthodontics and Dentofacial Orthopedics, Graduate School of Dentistry, Osaka University, 1-8 Yamada-Oka, Suita, 565-0871, Japan.

Phone: +81-6-6879-2958

Fax: +81-6-6879-2960

Email: sugihara.maiko.dent@osaka-u.ac.jp

Yuji Mishina, PhD

Department of Biologic and Materials Sciences & Prosthodontics, University of Michigan School of Dentistry, Ann Arbor, USA

4222A Dental, 1011 N. University Ave, Ann Arbor, MI 48109-1078, USA

Phone: 734-763-5579

Fax: 734-647-2110

E-mail: mishina@umich.edu

**Supplementary Figure 1.** The body weights of each group. The body weights of the 3-week group (**A**), 9-week group (**B**), 12-weekgroup (**C**), and the 9-week group fed either a hard diet (HD) or a soft diet (SD) for 6 weeks (**D**) were measured at the end of the experiment. n = 7 (3-week), 10 each (9-week), 10 each (12-week), 5 for control+HD, cKO+HD, control+SD, and cKO+SD.


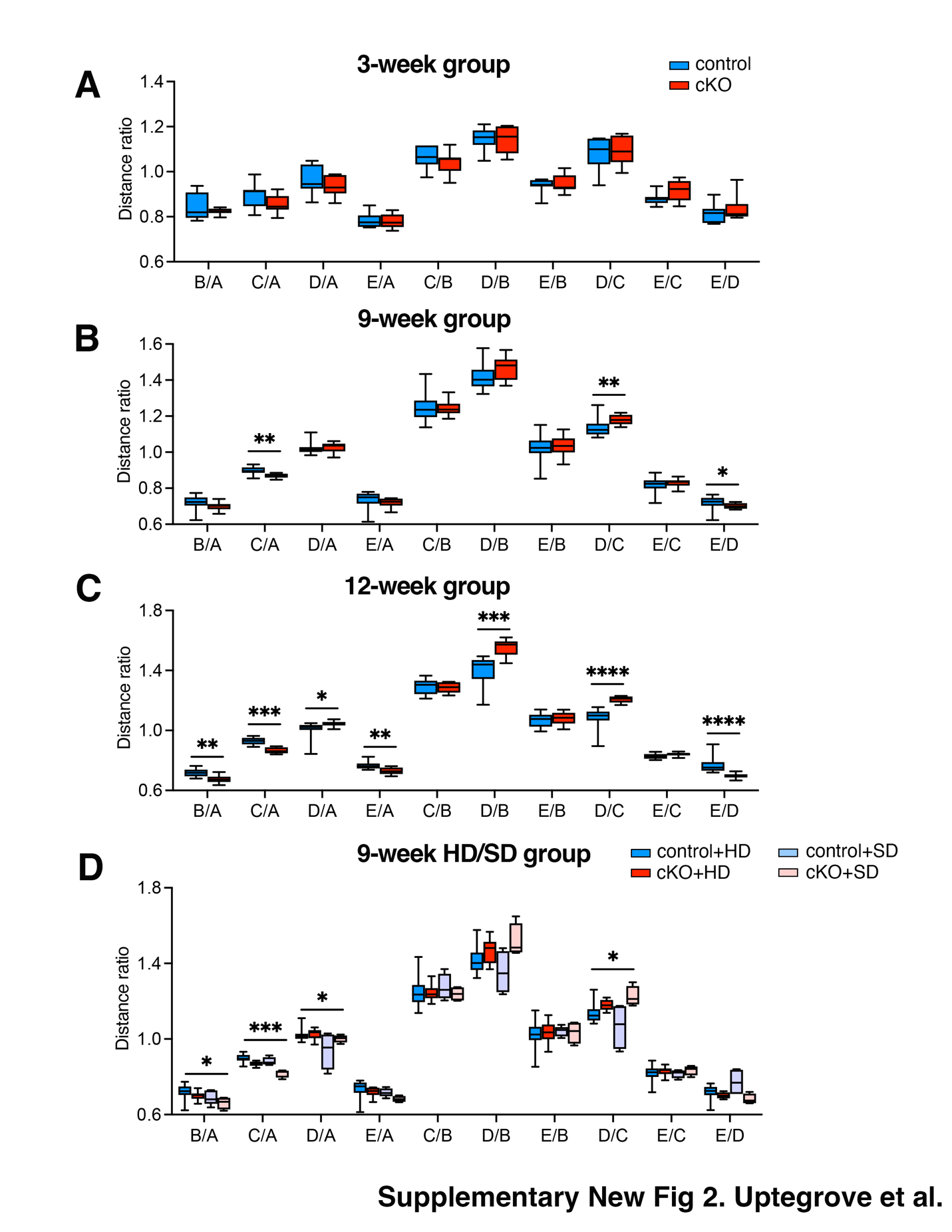


**Supplementary Figure 2.** The ratios of the linear measurements in each group. The ratios of the posterior length (A), anterior length (B), ascending height (C), descending height (D), and posterior height (E) were analyzed in the 3-week group (**A**), 9-week group (**B**), 12-week group (**C**), and the 9-week group fed either a hard diet (HD) or a soft diet (SD) for 6 weeks (**D**). The detailed length measurements are described in Fig. 2B. n = 7 (3-week), 10 (9-week), 10 (12-week), 5 each for control+HD, cKO+HD, control+SD, and cKO+SD. *p<0.05, **p<0.01, ***p<0.001, ****p<0.0001.


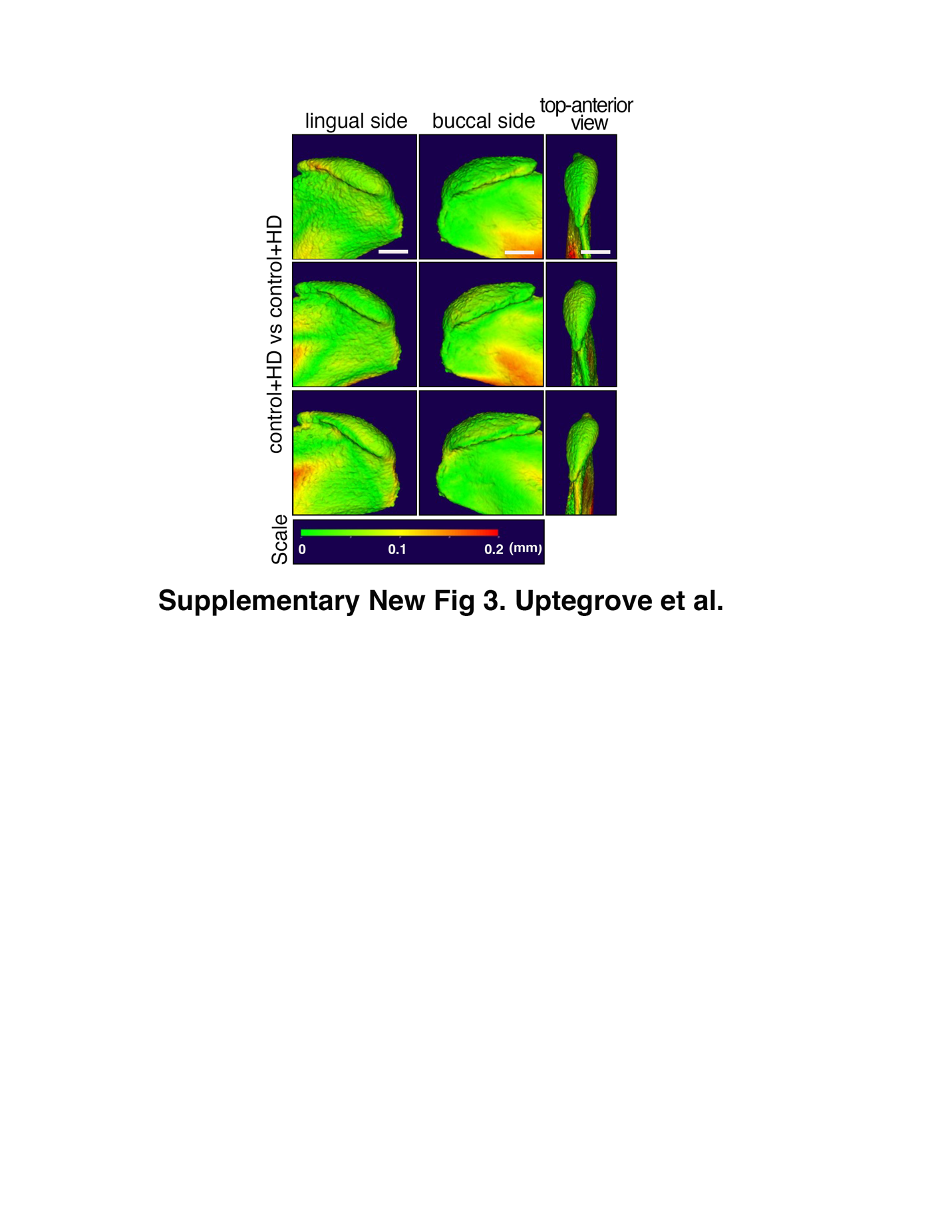


**Supplementary Figure 3.** Representative superimposed images of the mandibular condylar head from control mice at 9 weeks are presented. Overlay images between one reference control and three different controls are displayed, demonstrating the consistency of the surface structures. The color gradient illustrates variances in bone surface distances between similar surface points. Green denotes morphological similarity, while yellow-to-red hues denotes reductions or increases in degree, respectively. Scale = 500 µm.
